# Analysis of cellular and molecular antitumor effects upon inhibition of SATB1 in glioblastoma cells

**DOI:** 10.1186/s12885-016-3006-6

**Published:** 2017-01-03

**Authors:** Anja Frömberg, Michael Rabe, Henry Oppermann, Frank Gaunitz, Achim Aigner

**Affiliations:** 1 Rudolf-Boehm-Institute for Pharmacology and Toxicology, Clinical Pharmacology, University of Leipzig, Haertelstrasse 16 – 18, D-04107 Leipzig, Germany; 2 Present address: Deptartment of Pediatrics, University Clinic Heidelberg, Heidelberg, Germany; 3 Department of Neurosurgery, University Hospital Leipzig, Leipzig, Germany

**Keywords:** SATB1, Glioblastoma, RNAi, siRNA, PEI nanoparticles

## Abstract

**Background:**

The Special AT-rich Sequence Binding Protein 1 (SATB1) regulates the expression of many genes by acting as a global chromatin organizer. While in many tumor entities SATB1 overexpression has been observed and connected to pro-tumorigenic processes, somewhat contradictory evidence exists in brain tumors with regard to SATB1 overexpression in glioblastoma and its association with poorer prognosis and tumor progression. On the functional side, initial data indicate that SATB1 may be involved in several tumor cell-relevant processes.

**Methods:**

For the detailed analysis of the functional relevance and possible therapeutic potential of SATB1 inhibition, we employ transient siRNA-mediated knockdown and comprehensively analyze the cellular and molecular role of SATB1 in glioblastoma.

**Results:**

In various cell lines with different SATB1 expression levels, a SATB1 gene dose-dependent inhibition of anchorage-dependent and –independent proliferation is observed. This is due to cell cycle-inhibitory and pro-apoptotic effects of SATB1 knockdown. Molecular analyses reveal SATB1 knockdown effects on multiple important (proto-) oncogenes, including Myc, Bcl-2, Pim-1, EGFR, β-catenin and Survivin. Molecules involved in cell cycle, EMT and cell adhesion are affected as well. The putative therapeutic relevance of SATB1 inhibition is further supported in an in vivo tumor xenograft mouse model, where the treatment with polymeric nanoparticles containing SATB1-specific siRNAs exerts antitumor effects.

**Conclusion:**

Our results demonstrate that SATB1 may represent a promising target molecule in glioblastoma therapy whose inhibition or knockdown affects multiple crucial pathways.

**Electronic supplementary material:**

The online version of this article (doi:10.1186/s12885-016-3006-6) contains supplementary material, which is available to authorized users.

## Background

Malignant glioblastoma is the most common primary adult brain tumor in Western nations [1]. Despite aggressive treatment regimens including surgery, chemo- and radiotherapy, the prognosis for patients with the highest grade tumor, glioblastoma multiforme (GBM), has remained very poor. In fact, the overall survival rate is 12–15 months and the 5-years survival rate is only 5%. Limitations in complete resection and resistance towards adjunct radio- and chemotherapy account for this failure of treatment strategies and demonstrate the need for other therapeutic approaches based on novel targets. An optimal candidate should be overexpressed in the tumor tissue, with less expression and relevance in normal tissue, and its inhibition should ideally lead to multiple cellular and molecular effects harmful to the tumor cell.

The Special AT-rich Sequence Binding Protein 1 (SATB1) has been shown to regulate the expression of a large number of genes by acting as a global chromatin organizer [2]. More specifically, SATB1 interacts with the altered sugar-phosphate backbone of the DNA, that is specific for double-stranded base-unpairing regions (BURs) often found in matrix attachment regions (MARs) at the base of chromatin loops [3]. In the nuclei of thymocytes, SATB1 has a cage-like network distribution and tethers specialized DNA sequences onto its network [4]. Additionally, SATB1 binds and connects so-called “chromatin-remodeling complexes” to DNA and thus functions as a “landing platform” for chromatin remodeling enzymes [5]. In this way, SATB1 folds chromatin into loops and allows SATB1 to control the expression of a multitude of genes in a manner that is dependent on cell type and cell function [4, 6–9]. SATB1 is required in some physiological processes including the development of thymocytes [7] and the activation of Th2 cells [6]; it furthermore participates in the development of epidermis and epidermal differentiation [10], in X-chromosome inactivation [11], cortical development [12] and in the differentiation of mouse embryonic stem cells [13].

More importantly, SATB1 has been found to be overexpressed in various tumors and associated with prognosis and clinicopathological features. Examples include aggressive breast cancer [2], gastric cancer [14, 15], prostate cancer [16], liver cancer [17], laryngeal squamous cell carcinoma [18], ovarian carcinoma [19, 20], cervical carcinoma [21], pancreatic carcinoma [22], colorectal cancer [23–27] and malignant melanoma [28]. In different tumor entities including breast cancer, small cell lung cancer, liver cancer, osteosarcoma, prostate cancer and colorectal cancer, the stable RNAi-mediated knockdown of SATB1 has revealed multiple effects on the cellular level, including cell cycle [17, 22, 26], cell proliferation [2, 17, 22, 25, 26, 29], apoptosis [17, 25, 29], epithelial-mesenchymal transition (EMT) [17], invasiveness [2, 16, 22, 25, 26, 29] and/or tumor growth [2, 16, 17, 26, 27, 29].

In brain tumors, the situation appears so far to be more complex. A significant association of SATB1 levels with histological grade and poor survival has been described in low and high grade astrocytoma including glioblastoma [29, 30]. According to the recent WHO classification, glioblastoma is defined as grade IV astrocytoma. Chu et al. demonstrated that SATB1 mRNA and protein expression was low in normal brain and in grade I-II astrocytoma specimens but highly upregulated in grade III-IV astrocytoma patients [31]. SATB1 expression was positively correlated with astrocytoma pathological grade, while a negative correlation with patients’ overall survival was found [31]. In contrast, another study found an inverse correlation between SATB1 expression and tumor grade/patient survival, and identified only phospho-SATB1 as relevant [32]. Likewise, a Rembrandt/TCGA database analysis (http://www.betastasis.com/glioma/rembrandt/gene_expression_in_glioma_subtypes/) did not support the notion of SATB1 overexpression in brain tumors. In another study, initial results in one cell line indicated a possible role of SATB1 in some cellular and molecular processes [29]. Despite opposite findings with regard to SATB1 expression in glioblastoma and tumor grade/prognosis, another study found inhibitory effects of a SATB1 decoy on cell proliferation and invasion [32].

Taken together, this clearly warrants a more detailed analysis of the molecular and cellular consequences of SATB1 inhibition in glioblastoma, in order to establish the functional relevance of different levels of SATB1 expression in this tumor and to evaluate the putative therapeutic value of SATB1 inhibition, beyond a therapeutically less relevant stable knockdown. In fact, in order to avoid possible adaptive processes upon constitutive knockdown or overexpression, we employed a transient siRNA-mediated knockdown strategy in this paper. We comprehensively analyze the cellular and molecular role of SATB1 in various glioblastoma cell lines with different SATB1 expression levels, establishing in vitro and in vivo the functional relevance of SATB1 in glioblastoma, and the possible therapeutic potential of SATB1 inhibition.

## Methods

### Cell lines, primary cultures and cell culture conditions

Glioblastoma cell lines T98G, U-87 MG, U373 and LN-229 were obtained from the American type culture collection (ATCC). MZ-54 and MZ-18 cell lines were kindly provided by Dr. Donat Kögel (Experimental Neurosurgery, Frankfurt University Clinic, Frankfurt, Germany) [33], and the G55T2 cell line was a kind gift from Dr. Katrin Lamszus (Dept. of Neurosurgery, University Medical Center Hamburg-Eppendorf, Hamburg, Germany) [34]. U343 cells were established by B. Westermark [35]. All cell lines were cultivated under standard conditions (37 °C, 5% CO_2_) in Iscove’s Modified Dulbecco’s Medium (IMDM; Sigma-Aldrich, St. Louis, MO), supplemented with 10% fetal calf serum (FCS) and 2 mM stable L-Alanyl-L-Glutamine (Biochrom GmbH, Berlin, Germany) unless stated otherwise. Depending on the cell lines and the experimental setup, appropriate plate sizes and cell densities were chosen to reach 80 to 90% cell confluency at the end of the experiment. Cell lines were regularly tested for (absence of) mycoplasma, using the Venor GeM kit (Biostep, Berlin, Germany) based on very sensitive PCR detection.

Primary cell cultures from surgically removed glioblastoma tissues were established as described [36]. Briefly, freshly removed tumor tissue was washed with PBS (phosphate buffered saline) and minced with a scalpel blade. After mincing, small tissue pieces were transferred to a 25 cm^2^ culture flask (TPP, Trasadingen, Switzerland) sprinkled with AmnioMax complete medium (Thermo Fisher Scientific, Darmstadt, Germany). Cells were incubated for 30 min at room temperature and finally, 1 ml AmnioMax complete medium was added. Incubation was then performed at 37 °C, 5% CO_2_ and humidified air in an incubator. Medium was changed after 72 h. As soon as a confluent layer was obtained, cells were removed from culture flasks by use of accutase (PAA, Pasching, Austria) and transferred to 75 cm^2^ culture flasks (TPP). AmnioMax Medium with AmnioMax Supplement was used for the first 2–3 weeks of cultivation. Thereafter, and in the experiments described, DMEM Medium supplemented with 2 mM Glutamax, streptomycin and penicillin and 10% fetal calf serum (Biochrome, Berlin, Germany) was used for cultivation.

### Analysis of SATB1 expression in primary glioblastoma and normal brain tissue

For the RNA isolation from primary tissue, fresh surgically obtained tumor tissue was transferred into RNAlater (Qiagen, Hilden; Germany) immediately after removal in order to stabilize RNA. Then, total RNA from 40 to 80 mg of stabilized tissue was extracted using the miRNeasy kit (Qiagen) and the RNA was stored at −80 °C until further use. For the isolation of mRNA from cultured cells and cell lines 0.5 × 10^6^ cells were used and also prepared using the miRNeasy kit according to manufacturer’s instructions. All patients provided written informed consent according to the German laws, as confirmed by the local ethics committee. Surgery was performed between 2010 and 2013 at the University of Leipzig, Medical Faculty, Department of Neurosurgery. The samples were histopathologically confirmed as glioblastoma multiforme. For cDNA synthesis the ImProm-II™ Reverse Transcription System (Promega, Mannheim, Germany) was employed according to manufacturer’s protocol, using 500 ng of total RNA. qRT-PCR was performed on a Rotor-Gene 3000 system (Qiagen) with SYBR Green (Maxima SYBR Green/ROX qPCR Master Mix, Thermo Scientific, Germany). Data analysis was performed using the Rotor-Gene 6 software (Version 6.1/Build 93; Corbett Research) and relative mRNA expression was calculated by the 2^-ΔCt^ method using TBP (TATA box binding protein) as housekeeping gene. cDNA from normal brain tissue was obtained from BioCat (Heidelberg, Germany).

### Transient transfection

SiRNAs were purchased from Sigma-Aldrich (Taufkirchen, Germany) or Eurofins MWG Operon (Ebersberg, Germany); see Additional file 1: Table S1 for sequence information. SiRNAs targeting luciferase (pGL3) were used as negative control. Prior to transfection, cells were seeded in appropriate cell culture plates and maintained overnight under standard conditions. 2.5 nM siRNA were transfected using INTERFERin™ (Polyplus, Illkirch, France), at 1 μl INTERFERin™/pmol siRNA (U-87 MG) or 0.5 μl INTERFERin™/pmol siRNA (G55T2, U343, MZ-18) according to the manufacturer’s protocol.

### RNA preparation and qRT-PCR in cell lines

Total RNA was isolated using TRI Reagent® (Sigma-Aldrich) according to manufacturer’s instructions. The RevertAid™ H Minus First Strand cDNA Synthesis Kit (Fermentas, St. Leon-Roth, Germany) was used to reversely transcribe 1 μg of total RNA with random hexamer primers. For quantitative PCR, a LightCycler® 2.0 (Roche, Mannheim, Germany) and the Absolute™ QPCR SYBR® Green Capillary Mix (Thermo Scientific) were used as described previously [37]. Quantification of gene expression was performed based on the ΔΔC_t_ method, with β-actin as reference housekeeping gene. Control experiments revealed that very similar results were obtained for β-actin vs. TBP as housekeeping genes, indicating the usefulness of both primer sets for normalization. Primers were purchased from Eurofins MWG Operon (for sequences, see Additional file 2: Table S2).

### Western blotting

5x10^4^ G55T2 or U-87 MG cells were seeded in 6-well plates and transfected as described above. 72 h (G55T2) or 96 h (U-87 MG) after transfection, cells were washed with PBS and lysed as described previously [37]. 50 μg (U-87MG) or 10–20 μg (G55T2) total protein was separated by SDS-PAGE, prior to transfer onto a 0.2 μM or 0.45 μM PROTRAN® nitrocellulose membrane (Whatman, Dassel, Germany). Membranes were blocked with 5% (w/v) non-fat dry milk in TBST (10 mM Tris/HCl, pH 7.6, 150 mM NaCl, 0.1% Tween 20), washed with TBST and incubated overnight with primary antibodies at 4 °C as detailed in Additional file 3: Table S3. After washing with TBST, membranes were incubated with horseradish peroxidase-coupled secondary antibodies (Additional file 3: Table S3) for 1 h at room temperature. Bound antibodies were visualized using the chemiluminescence ECL kit from Thermo Scientific. For parallel detection of phosphorylated proteins and their corresponding unphosphorylated counterpart, membranes were incubated in stripping buffer (0.2 M glycin, 3.5 mM sodium dodecyl sulfate, 1% (v/v) Tween-20, pH 2.2) for 30 min at room temperature, washed with TBST and blocked again with 5% (w/v) non-fat dry milk in TBST, prior to further processing as described above.

### Anchorage-dependent and -independent proliferation

Anchorage-dependent proliferation was analyzed using a WST-1 colorimetric assay (Roche). 200 cells/well were seeded in 96-well plates and transfected as described above. At the time points indicated in the Figures, viable cells were quantified in triplicate wells using WST-1 colorimetric assay according to manufacturer’s protocol. To measure anchorage-independent proliferation, U-87 MG cells were seeded in 6-well plates and transfected as described above. 48 h after transfection, soft agar assays were performed as described previously [37]. Soft agars were run in triplicate wells and incubated under standard conditions. At the time points indicated, colonies > 50 μm were counted by at least two blinded investigators.

### Cell cycle analysis

For cell cycle analysis, 1×10^4^ U-87 MG or G55T2 cells were seeded into 24-well plates and transfected as described above. 72 h after transfection, cells were treated with 100 ng/ml nocodazole (Merck-Calbiochem®, Darmstadt, Germany) in IMDM/10% FCS for 8 h to induce a G2/M arrest. The cells were harvested by trypsinization, washed with PBS and fixed with 70% ethanol at 4 °C overnight. Prior to addition of 50 μg/ml propidium iodide (Sigma-Aldrich), cells were incubated with 50 μg/ml RNase A for 30 min at 37 °C and subsequently analyzed by flow cytometry using an Attune® Acoustic Focusing Cytometer (Life Technologies, Darmstadt, Germany).

### Apoptosis assays

To quantify the activity of caspases 3 and 7, the bioluminescent Caspase-Glo® 3/7 assay (Promega, Mannheim, Germany) was used. 300 cells (U-87 MG) or 750 cells (G55T2) were seeded per well in 96-well plates, transfected as described above and maintained under standard conditions for 96 h. The Caspase-Glo® assay was performed according to the manufacturer’s protocol. Luminescence was measured using a POLARstar Omega reader (BMG Labtec, Jena, Germany) after 1 h of incubation at room temperature in the dark. A WST-1 assay was performed in parallel on the same plate as described above, to normalize for slight variations in cell densities.

### Mouse xenograft model

To investigate the effects of RNAi-mediated SATB1-knockdown on tumor growth in vivo, 3 ×10^6^ U-87 MG cells in 150 μl PBS were injected into both flanks of 6–8 weeks old athymic nude mice (Crl:CD1-Foxn1nu, Charles River Laboratories, Sulzfeld, Germany). When solid tumors were established, mice were randomized into treatment and control groups. The tumors were treated with intratumoral injections of 2 μg siRNA complexed with 10 μg PEI F25-LMW [38] in a total volume of 30 μl. The tumors were treated every 2 to 3 days and tumor growth was monitored as indicated in Fig. 4a. Animal studies were conducted according to the national regulations of animal welfare and approved by the local authorities (Regierungspräsidium Giessen, Germany).

### Statistics

Statistical analysis was performed by Student’s t-test and significance levels are * = *p* < 0.05, ** = *p* < 0.01, *** = *p* < 0.001, # = not significant as compared to siCtrl, unless indicated otherwise. Values are shown as means +/− s.e.m.

## Results

### Determination of SATB1expression in primary glioblastoma tissue and cells, compared to normal brain tissue

In contrast to other tumor entities where SATB1 upregulation as compared to normal tissue has been well established, the situation in glioblastoma appears less clear (see Background). Therefore, we first analyzed SATB1 mRNA levels of ten different primary tumor samples. While all tumors showed SATB1 expression, levels varied considerably between different samples, with a maximum ~10-fold difference (Additional file 4: Figure S1, center). The same was true for primary tumor cells derived from these tumors, with values often, but not in all cases being comparable between a primary tumor and its corresponding primary cell line (Additional file 4: Figure S1, center). Notably, in comparison to normal brain tissue no SATB1 upregulation was observed in tumors, with tumor levels rather being even lower (Additional file 4: Figure S1, left). From these data, we conclude that SATB1 expression levels may only poorly predict its functional relevance, thus requiring more detailed analyses in a panel of cell lines with different SATB1 expression levels.

### Expression of SATB1 in various glioblastoma cell lines and comparison to SATB2

Based on the heterogeneous situation with regard to SATB1 expression levels in glioblastoma, we screened a set of eight commercially available and well-established glioblastoma cell lines for SATB1 levels. qRT-PCR results demonstrated substantial expression of SATB1 in 7/8 cell lines, with the only exception being T98G cells that showed almost no SATB1 (Additional file 4: Figure S1, right). Some variations between positive cell lines were observed with a maximum ~9-fold difference in SATB1. SATB2, which is considered as a functional counterpart of SATB1, was analyzed as well. Here, expression was observed in all 8 cell lines (Additional file 5: Figure S2). The comparison between SATB1 and SATB2 levels revealed no correlation in expression levels.

**Fig. 1:**
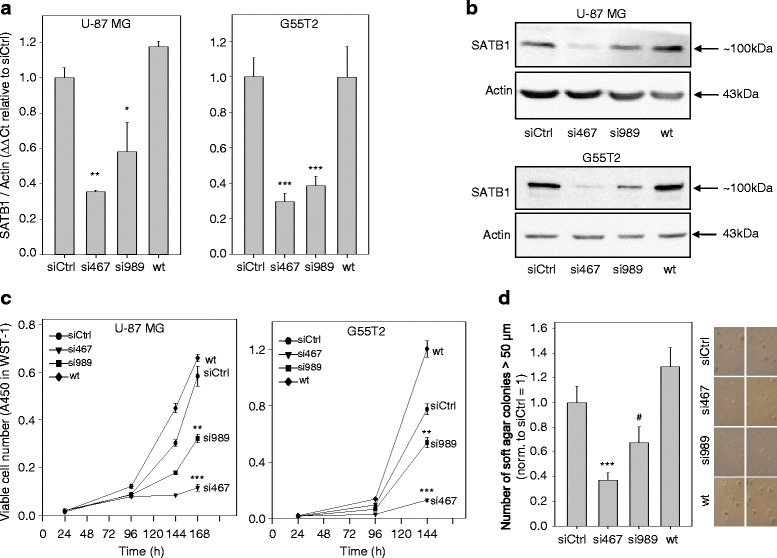
siRNA-mediated SATB1 knockdown exerts tumor cell inhibitory effects. SATB1 knockdown upon transfection of SATB1-specific siRNAs si467 or si989 in U-87 MG and G55T2 glioblastoma cells, as determined on (**a**) mRNA (*n* = 3–4 experiments, performed in duplicates and analyzed 72 h after transfection) and (**b**) protein level (*n* = 4 experiments, analyzed 96 h after transfection, one representative shown). Actin was used as loading control, and transfections with siRNAs targeting the irrelevant protein luciferase served as negative controls (siCtrl). Two specific siRNAs were explored, with si467 being more efficient than si989. **c** Marked inhibition of anchorage-dependent proliferation, particularly when using the more potent si467. **d** Decreased colony formation ability of U-87 MG cells in soft-agar assay, indicative of impaired anchorage-independent growth (right panel: representative photos of soft-agar colonies)

The expression of SATB1 in almost all glioblastoma cell lines provided the basis for subsequent functional studies. To this end, four glioblastoma cell lines (U-87 MG, MZ-18, G55T2 and U343) with high or low SATB1 levels, thus covering the broad range of SATB1 expression, were selected for transient RNAi-mediated knockdown. To exclude false-positive results due to off-target effects and to allow the establishment of gene-dose effects, two siRNAs validated previously for specific SATB1 knockdown to different degrees were employed and compared to untreated as well as to negative control transfected cells (siRNA targeting the luciferase gene which is not expressed in glioblastoma cells). In all cell lines, qRT-PCR after single transfection with the less potent SATB1-specific siRNA (si989) revealed a ~ 50% SATB1 knockdown in comparison to negative controls (wt and siCtrl). A > 60% knockdown was observed with the more potent si467 (Fig. 1a and Additional file 6: Figure S3A), with only minor differences between the four selected cell lines. Knockdown results were confirmed on the protein level by Western blots, showing a concomitant reduction of SATB1 with bands upon si467 transfection being close to the limit of detection (Fig. 1b). This was also true at later time points (e.g., 120 h, 144 h after transfection; data not shown). We thus concluded that the transient siRNA transfection provides an efficient tool for specific SATB1 downregulation.

### Inhibitory effects of SATB1 knockdown on cell proliferation

To initially explore the effects of SATB1 knockdown on overall cell proliferation and viability, WST-1 proliferation assays were performed. Growth curves revealed a marked reduction of cell proliferation upon transfection with si989 in all cell lines (Fig. 1c and Additional file 6: Figure S3B). Except for G55T2 cells, no nonspecific transfection effects were observed. Using the more potent si467, cell proliferation was reduced by > 80%, indicating very profound effects of the SATB1 knockdown on the number of viable cells. These results were confirmed in a soft agar assay, which resembles more closely the in vivo situation. Upon siRNA transfection of U87 MG cells, a reduction in the anchorage-independent colony formation was observed, which was again dependent on the siRNA efficacy and reached a > 60% decrease in colonies in the case of si467 (Fig. 1d).

### Cell cycle inhibition and induction of apoptosis upon SATB1 knockdown

**Fig. 2:**
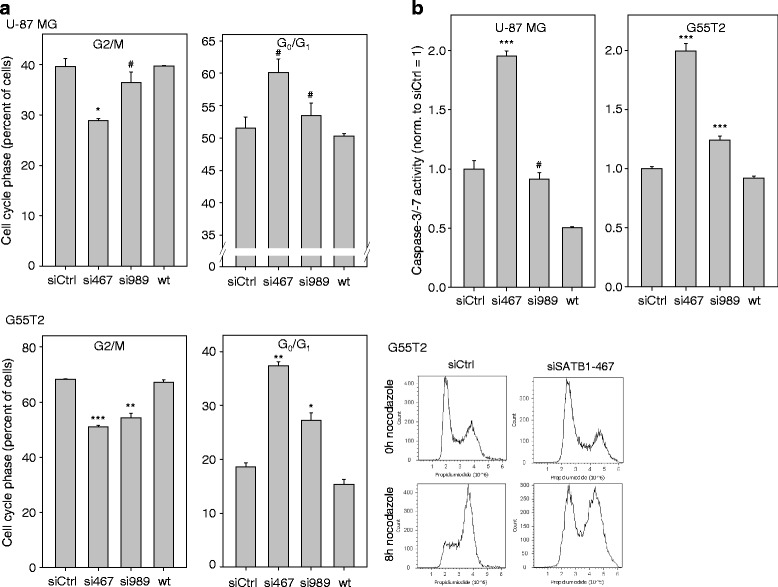
Effects of SATB1 knockdown on cell cycle and apoptosis. **a** Transient SATB1 knockdown leads to a decreased percentage of cells in G2/M and an increase of cells in G_0_/G_1_, as determined in U-87 MG (upper panel) and G55T2 glioblastoma cells (lower panel). Cell cycle inhibition is more pronounced when using the more efficient si467, indicating a SATB1 gene-dose effect. Measurements were performed 8 h after addition of nocodazole (see text for details; *n* = 2 experiments per cell line). Lower right: representative flow cytometry histograms. **b** Induction of apoptosis upon transient SATB1 knockdown, as determined by increased caspase-3/-7 activity. Effects are seen in both U-87 MG (left) and G55T2 glioblastoma cells (right) and are dependent on the degree of SATB1 reduction

To further explore the underlying cellular mechanisms of the reduction of the number of viable cells upon SATB1 knockdown, we next analyzed effects on cell cycle. Here and in subsequent experiments, we selected the two cell lines, U-87 MG and G55T2. Cells were transfected with the respective siRNAs and 72 h later nocodazole treatment was started in order to implement a G2/M block. When cells were propidium iodide-stained and analyzed by flow cytometry upon 8 h nocodazole treatment, 40% (U87 MG) or 70% (G55T2) of the cells were in G2/M (Fig. 2a). In contrast, upon transfection with SATB1-specific siRNAs si989 and especially si467 this percentage was reduced, indicative of cell cycle deceleration/arrest in G_0_/G_1_ with a smaller number of cells reaching the block within the selected time frame. Consequently, a larger fraction of cells was determined in the G_0_/G_1_ phase (Fig. 2a, right panels). In both cell lines, the degree of cell cycle inhibition was dependent on the siRNA efficacy and thus the residual SATB1 levels, with si467 showing more profound effects.

In addition to cell cycle deceleration, SATB1 knockdown led to the induction of apoptosis as indicated by increasing caspase-3/-7 activity. More specifically, while the transfection with si989 led to little or no effects depending on the cell line, the more potent si467 resulted in a profound up to 2-fold increase in caspase activity.

### Molecular consequences of SATB1 knockdown

**Fig. 3:**
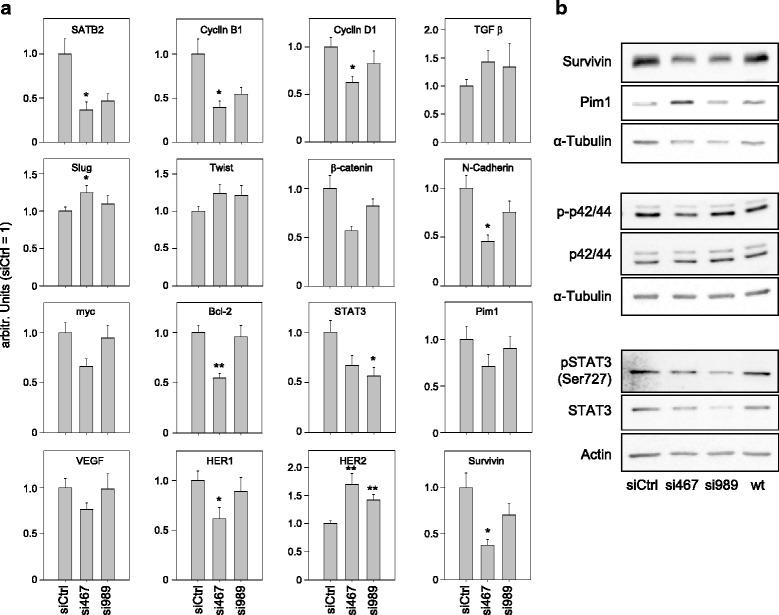
Analysis of molecular consequences of SATB1 knockdown in G55T2 cells. The siRNA-mediated knockdown of SATB1 affects the expression levels of a broad spectrum of (proto-) oncogenes and various molecules involved in cell cycle, EMT, signal transduction and cell adhesion, as determined on the mRNA level by quantitative RT-PCR at 48 – 72 h after transfection (**a**) and on the protein level by Western blotting (**b**). For details, see text. In (**a**), differences that reached significance are indicated (*n* = 5–6 experiments determined at 72 h after transfection; Slug and Twist: *n* = 9 experiments determined at 48 – 72 h after transfection)

The siRNA-mediated knockdown of SATB1 revealed effects on the expression levels of a broad spectrum of (proto-) oncogenes and various molecules involved in cell cycle, EMT, signal transduction and cell adhesion, as detected on the mRNA level by quantitative RT-PCR and on the protein level by Western blotting in G55T2 cells. Although the other family member, SATB2, is considered as a potential functional counterpart with opposite roles, qRT-PCR revealed it was downregulated by siRNAs 467 and 989 in parallel with SATB1 (Fig. 3a). By analyzing the siRNA sequences with regard to sequence homologies, it was firmly excluded that this observation was due to unwanted off-target effects of SATB1-specific siRNAs on SATB2 based on any partial sequence homology [39]. Additionally, the observed absence of decreased SATB2 levels upon SATB1 knockdown in cells from another tumor entity further substantiates the notion of SATB2 reduction as a specific effect downstream of SATB1. In line with the observed cell cycle deceleration, cell cycle proteins Cyclin B1 and D1 that are often overexpressed in tumor cells were downregulated upon SATB1 knockdown. Again, the transfection with the more potent si467 led to a more profound reduction of Cyclin mRNAs with a > 50% decrease in the case of Cyclin B1. Rather mild effects were observed on TGFβ or the transcription factors Slug and Twist, with slightly increased mRNA levels upon SATB1 knockdown. In contrast, profound inhibitory effects were detected on the cell adhesion and gene transcription regulating proto-oncogene β-catenin and on N-Cadherin which were again dependent on the siRNA efficacy and led to ~50% reduced levels in the case of si467. While si989 exerted effects as well, albeit to a lesser degree, in the case of Myc and Bcl-2 downregulated mRNA levels were only observed with si467, indicative of a threshold of minimally required SATB1 knockdown. The same was true for the (rather mild) reduction in mRNA levels of the pro-angiogenic VEGF, while in the case of the proto-oncogenes Pim1 and HER1 si989 slightly reduced mRNA expression while si467 led again to a more profound decrease up to 60–70% residual level. Opposite to HER1, the SATB1 knockdown led to an increase in HER2 expression which was again gene-dose dependent and thus most profound upon si467 transfection. Finally, profound inhibitory effects on the mRNA level were also observed on STAT3 and Survivin (Fig. 3a). The latter finding correlated well with decreased protein levels of the anti-apoptotic protein Survivin as determined in Western blot experiments, with si467 showing the most profound effects (Fig. 3b, upper panel). Interestingly, the same siRNA led to an increase, rather than decrease, in Pim1 protein levels. Consequences of SATB1 knockdown were also explored with regard to downstream signaling. While the total expression of p42/44 (ERK1/2) remained unchanged, ERK phosphorylation was reduced (Fig. 3b, center panel). Again, this effect was only observed upon si467 transfection indicative of the requirement of sufficiently profound SATB1 knockdown. Quite in contrast, effects of SATB1 knockdown on STAT3 were already observed on the level of protein expression (Fig. 3b, lower panel), thus being in line with the qRT-PCR data, with a concomitant and parallel decrease of phospho-STAT3.

### Tumor inhibitory effects of SATB1 knockdown in vivo

**Fig. 4:**
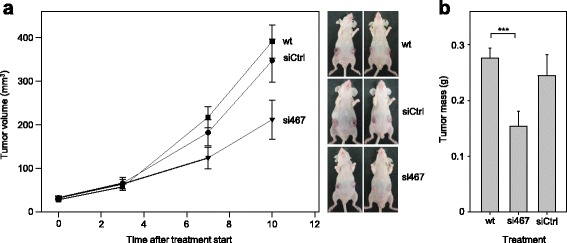
Inhibition of tumor growth *in vivo* upon SATB1 knockdown. **a** Subcutaneous U-87 MG tumor xenografts were established in athymic nude mice. Upon randomization, mice were treated by i.t. injection of 2 μg siRNAs specific for SATB1 (si467) vs. negative control siRNAs (siCtrl). For siRNA delivery, siRNAs were formulated in polymeric nanoparticles based on a low-molecular weight polyethylenimine (PEI F25-LMW). Untreated mice (‘wt’) served as additional negative control for the absence of non-specific treatment effects. Right: representative pictures of mice (*n* = at least 13 tumor xenografts per group). **b** The determination of masses of the tumor xenografts explanted upon termination of the experiment confirmed the tumor growth inhibition

The consequence of siRNA-mediated SATB1 knockdown was finally tested in a more relevant in vivo situation by exploring tumor-inhibitory effects in an s.c. xenograft model. Rather than using stably transfected cells, which may well interfere with tumor xenograft formation, the SATB1 knockdown was performed in already established tumors. To this end, mice were treated with siRNAs formulated in polymeric, polyethylenimine (PEI)-based nanoparticles, which mediate siRNA protection, cellular delivery and intracellular release. As shown previously by our group, PEI/siRNA nanoparticles allow for the knockdown of the respective target gene (see e.g. [40, 41]). Indeed, a ~ 40% inhibition of the growth of established tumors was observed as compared to untreated or PEI/negative control siRNA-treated mice (Fig. 4a). Results from tumor size measurements were paralleled and confirmed by a reduction of tumor mass, as detected upon termination of the experiment by excision of the tumor xenografts for weight determination (Fig. 4b).

## Discussion

In the light of conflicting results regarding a positive [29–31] or negative [32] correlation between SATB1 expression and clinicopathological features of glioblastoma, and thus the relevance of SATB1 in these tumors, a deeper understanding of the cellular and molecular roles of SATB1 in glioblastoma cells is required. Indeed, our qRT-PCR screening data presented here do not support the notion of SATB1 overexpression in glioblastoma. While a recent analysis in colorectal cancer [42] has identified divergent expression patterns of SATB1 on the mRNA and protein level versus normal tissues, which may offer an explanation for rather low mRNA levels in tumors, only in-depth functional studies allows for evaluating the relevance of SATB1 in glioblastoma.

Here, the approach of transient RNAi, avoiding issues related to stable cell transfection with constitutive knockdown or overexpression, offers an excellent avenue. Notably, we found proliferation inhibitory effects of SATB1 knockdown in *all* glioblastoma cell lines tested, thus being independent of initial SATB1 expression levels. This emphasizes the general relevance of SATB1 beyond differences in mRNA levels and suggests SATB1 inhibition as a promising therapeutic avenue. Subsequent cellular analyses also revealed that this tumor cell inhibition upon SATB1 knockdown is based on the induction of apoptosis, as indicated previously [29], but also on cell cycle deceleration. This supports the notion that SATB1 acts on several pathways and its inhibition thus exerts multiple effects in parallel. In line with this, various key players are affected by SATB1 knockdown on the molecular level. This includes the downregulation of Cyclins B1 and D1 (deceleration of cell cycle in the transition from G2 to M and from G1 to S, respectively), the activation of caspase-3/-7 (induction of apoptosis) and the decrease in the pro-survival protein Survivin. The latter finding supports our previous studies in colon carcinoma [39, 43] suggesting a SATB1 – Pim1 – Survivin axis that leads to a parallel Survivin decrease upon SATB1 knockdown. However, we found Pim1 protein levels being even elevated instead. This indicates that Pim1 expression is not merely determined on the level of transcription, but that the Pim1 protein is also post-transcriptionally regulated and subject to degradation/stabilization as described previously ([44] and references therein).

Our results furthermore support the previous hypothesis [29] that SATB1 knockdown may also affect the anti-apoptotic proto-oncogene Bcl-2. Here like in the case of another important proto-oncogene, Myc, it should be noted that, when comparing siRNAs with different SATB1 knockdown efficacies, a knockdown below a certain threshold is required for altering on Myc or Bcl-2 mRNA levels. Thus, while our knockdown studies reveal a ‘SATB1 gene dose effect’ on the expression of many genes (e.g., Cyclin B1, Cyclin D1, N-Cadherin, β-catenin, Survivin, HER2), other mRNAs (Myc, Bcl-2, VEGF, HER1) are only affected by very profound SATB1 inhibition (si467). Alterations are also seen in the transcription factor STAT3. At first glance, the observed differences in STAT3 phosphorylation could be attributed to HER1 (EGFR) downregulation with subsequently decreased EGFR-STAT3 signaling (as previously shown to be relevant for example in peripheral nerve sheath tumors [45]). It should be noted, however, that here differences in band intensities actually reflect differences in STAT3 expression, as shown on mRNA and protein level. To the contrary, the observed decrease in phospho-p42/44 (p-ERK 1/2) rests on reduced phosphorylation rather than differences in expression. The downregulation of HER1 (EGFR) upon SATB1 knockdown is in line with previous studies in other tumor entities [2, 39]. Interestingly, however, this is not true for another member of the EGFR family, HER2, where upregulation rather than downregulation is observed, thus suggesting activation rather than inactivation of an oncogene upon SATB1 knockdown. Since previously direct effects of SATB1 on HER2 have been shown [2], one explanation for this discrepancy may be mutual effects of one HER receptor (here: EGFR) on the expression of other family members (here: HER2), as found in other tumor entities (Gutsch and Aigner, unpublished). This also demonstrates that the functional relevance and molecular effects of a given target gene (in this case SATB1) need to be evaluated in the precise tumor context.

The EGFR pathway is one of the most significant signaling pathways in glioblastoma, and EGFR is among the major genetic factors affecting the pathogenesis and prognosis of GBM. This emphasizes the relevance of SATB1 knockdown on reducing EGFR expression. Another central player in glioblastoma is β-catenin which has been found overexpressed for example in astrocytic tumors and correlated with poor prognosis and short patient survival [46, 47]. Here we describe β-catenin downregulation upon SATB1 knockdown. While activating mutations are not prevalent in glioblastoma, it was shown previously that proliferation of several glioblastoma cells could be significantly inhibited by siRNA-mediated targeting of β-catenin [48]. Thus, β-catenin downregulation may well contribute to the observed inhibitory effects of SATB1 inhibition. Our findings are also in line with a recent study in colorectal cancer, where SATB1 was found to be a target of Wnt/β-catenin signaling while in turn simultaneously regulating β-catenin expression [27]. Taken together, this establishes a SATB1 knockdown effect on two central factors in glioblastoma, EGFR and β-catenin.

Finally, inhibitory effects of SATB1 knockdown were also observed on two molecules relevant in other important processes. While the very profound effect on in N-Cadherin expression connects SATB1 expression with tumor cell motility and invasiveness, it should be noted that in high grade glioblastomas N-Cadherin has been found to be inversely correlated with invasive behavior [49]. This suggests that the N-Cadherin decrease observed here upon SATB1 knockdown may rather enhance invasive properties. On the other hand, albeit downregulated to a lesser extent, the reduction of VEGF provides a molecular explanation for the previous finding that SATB1 inhibition leads to anti-angiogenesis [29].

## Conclusion

The transient knockdown approach chosen here reflects a therapeutic situation and, by using an siRNA delivery system based on polymeric nanoparticles developed in our lab, can also be employed in vivo. This allowed to study SATB1 knockdown in established tumors, thus clearly distinguishing inhibitory effects of SATB1 knockdown on tumor growth from just reducing tumor cell grafting. In light of this, and considering the multiple effects of targeting SATB1, the observed tumor-inhibitory effects are very promising with regard to future therapeutic implications. Our findings, also in the context of previous studies, provide a basis for the explanation of the observed antitumor effects on the cellular and molecular level.

## Additional files

Additional file 1: Table S1. Sequences of siRNAs used in this study. (PDF 4 kb)
Additional file 2: Table S2. Primers used in this study. (PDF 12 kb)
Additional file 3: Table S3. Primary and secondary antibodies used for Western blotting in this study, and the specifications of the buffers used for dilution. (PDF 11 kb)
Additional file 4: Figure S1. SATB1 mRNA levels in normal brain tissue versus primary glioblastoma tissue and primary cell lines derived thereof, and established cell lines. Expression levels were determined by qRT-PCR. Denominations of the primary material on the x-axis refer to patients’ IDs. (PDF 12 kb)
Additional file 5: Figure S2. Expression of SATB2 in various glioblastoma cell lines, as determined on the mRNA level by qRT-PCR. (PDF 4 kb)
Additional file 6: Figure S3. siRNA-mediated SATB1 knockdown and tumor cell inhibition. (A) SATB1 knockdown upon transfection of SATB1-specific siRNAs si467 or si989 in U343 and MZ-18 glioblastoma cells, as determined on mRNA level (*n* = 2–3 experiments performed in duplicates and analyzed 72 after transfection). Actin was used as loading control. (B) Marked inhibition of anchorage-dependent proliferation, particularly when using the more potent si467. (PDF 14 kb)

## Abbreviations

GBM: Glioblastoma multiforme
IMDM: Iscove’s Modified Dulbecco’s Medium
PBS: Phosphate-buffered saline
qRT-PCR: Quantitative real-time polymerase chain reaction
SATB1: Special AT-rich sequence binding protein 1
SDS: Sodium dodecyl sulfate
siRNA: Small interfering RNA
