# Supplementary material for: Analysis of cellular and molecular antitumor effects upon inhibition of SATB1 in glioblastoma cells
Source: BMC Cancer. 2017 Jan 3;17:3. doi: 10.1186/s12885-016-3006-6 (PMC5209874; doi:10.1186/s12885-016-3006-6)
Supplement: Additional file 1: Table S1. — Sequences of siRNAs used in this study. (PDF 4 kb) [file 12885_2016_3006_MOESM1_ESM.pdf]

Additional file 1: Table S1. Sequences of siRNAs

**Sequences of siRNAs targeting SATB1 mRNA:**		
si467	(sense)	5’- GCU UCA AGA UGU GUA UCA UdTdT -3’
	(antisense)	5’- AUG AUA CAC AUC UUG AAG CdTdT -3’
si989	(sense)	5’- GUA UGC AGU GAA UAG ACU UdTdT -3’
	(antisense)	5’- AAG UCU AUU CAC UGC AUA CdTdT -3’
**Sequences of siRNAs targeting firefly luciferase mRNA (negative control, siCtrl):**		
siLuc3	(sense)	5’- CUU ACG CUG AGU ACU UCG AdTdT -3’
	(antisense)	5’- UCG AAG UAC UCA GCG UAA GdTdT -3’
