# Supplementary material for: Analysis of cellular and molecular antitumor effects upon inhibition of SATB1 in glioblastoma cells
Source: BMC Cancer. 2017 Jan 3;17:3. doi: 10.1186/s12885-016-3006-6 (PMC5209874; doi:10.1186/s12885-016-3006-6)
Supplement: Additional file 2: Table S2. — Primers used in this study. (PDF 12 kb) [file 12885_2016_3006_MOESM2_ESM.pdf]

**cDNA primer sequences (5’ to 3’):**					
Bcl-2	forward	ATG TGT GTG GAG AGC GTC AA	SATB1	forward	CGA TGA ACT GAA ACG AGC AG
	reverse	GGG CCG TAC AGT TCC ACA AA		reverse	CGG AGG ATT TCT GAA AGC AA
β-actin	forward	CCA ACC GCG AGA AGA TGA	SATB2	forward	GCG ACA GTG GCC GAC ATG CT
	reverse	CCA GAG GCG TAC AGG GAT AG		reverse	GGT TCC ACT GCT CCG CAG GC
β-Catenin	forward	TCG AGG ACG GTC GGA CTC CC	Slug	forward	GAA CTC ACA CGG GGG AGA AGC C
	reverse	CTG CCA GTG ACT AAC AGC CGC		reverse	AGA ATG GGT CTG CAG ATG AGC CCT
Cyclin B1	forward	CAT GGT GCA CTT TCC TCC TT	STAT3	forward	GAG GAC TGA GCA TCG AGC A
	reverse	AGG TAA TGT TGT AGA GTT GGT GTC		reverse	CAT GTG ATC TGA CAC CCT GAA
Cyclin D1	forward	CCT CGG TGT CCT ACT TCA AA	Survivin	forward	TGA TGA GAG AAT GGA GAC AGA G
	reverse	CAC TTC TGT TCC TCG CAG A		reverse	ACA GCA GTG GCA AAA GGA G
HER1	forward	ACA CAG AAT CTA TAC CCA CCA GAG T	TGFβ	forward	CAA CAA TTC CTG GCG ATA CCT
	reverse	ATC AAC TCC CAA ACG GTC AC		reverse	GCT AAG GCG AAA GCC CTC AAT
HER2	forward	TGG CTC AGT GAC CTG TTT TG	TBP	forward	TTG ACC TAA AGA CCA TTG CAC
	reverse	GGT CCT TAT AGT GGG CAC AGG		reverse	GCT CTG ACT TTA GCA CCT GTT
myc	forward	CAC CAG CAG CGA CTC TGA	Twist	forward	GAC CTA GAT GTC ATT GTT TCC AG
	reverse	GAT CCA GAC TCT GAC CTT TTG		reverse	TTA GTT ATC CAG CTC CAG AGT
N-Cadherin	forward	AAC TGG GCC AGG AGC TGA CCA	VEGF	forward	GGA AAC CAG CAG AAA GAG GA
	reverse	GTG CCC TCA AAT GAA ACC GGG CT		reverse	GTC ACT CAC TTT GCC CCT GT
Pim1	forward	ATC AGG GGC CAG GTT TTC T			
	reverse	GGG CCA AGC ACC ATC TAA T			

Frömberg et al Additional file 2: Table S2
