# Supplementary material for: Analysis of cellular and molecular antitumor effects upon inhibition of SATB1 in glioblastoma cells
Source: BMC Cancer. 2017 Jan 3;17:3. doi: 10.1186/s12885-016-3006-6 (PMC5209874; doi:10.1186/s12885-016-3006-6)
Supplement: Additional file 3: Table S3. — Primary and secondary antibodies used for Western blotting in this study, and the specifications of the buffers used for dilution. (PDF 11 kb) [file 12885_2016_3006_MOESM3_ESM.pdf]

Additional file 3: Table S3. Antibodies and antibody dilution conditions

**Primary Antibodies for Western Blotting:**		Diluted in
rabbit anti-actin	Santa Cruz, Heidelberg, Germany	3% (w/v) non-fat dry milk in TBST
mouse anti-α-Tubulin	Sigmal Aldrich, St. Louis, MO	3% (w/v) non-fat dry milk in TBST
rabbit anti-phospho-p44/42 MAPK	Cell Signaling, Danvers, MA	5% BSA (w/v) in TBST
rabbit anti-p44/42 MAPK	Cell Signaling, Danvers, MA	5% BSA (w/v) in TBST
rabbit anti-Pim1	Epitomics, Burlingame, CA	3% (w/v) non-fat dry milk in TBST
rabbit anti-SATB1 (EPR3951)	Epitomics, Burlingame, CA	5% (w/v) non-fat dry milk in TBST
rabbit anti-phospho-STAT3 (ser727)	Cell Signaling, Danvers, MA	5% BSA (w/v) in TBST
mouse anti-STAT3	Pierce, Thermo Fisher Scientific, Rockford, IL	5% (w/v) non-fat dry milk in TBST
rabbit anti-Survivin	Epitomics, Burlingame, CA	3% (w/v) non-fat dry milk in TBST
**Secondary Antibodies for Western Blotting:**		
goat anti-rabbit IgG, HRP-linked	Cell Signaling, Danvers, MA	3% (w/v) non-fat dry milk in TBST
sheep anti-mouse IgG, HRP-linked	GE Healthcare, Freiburg, Germany	3% (w/v) non-fat dry milk in TBST
TBST	10 mM Tris/HCl, pH 7.6, 150 mM NaCl, 0.1% Tween 20	
