# Supplementary figures and images for: Analysis of cellular and molecular antitumor effects upon inhibition of SATB1 in glioblastoma cells
Source: BMC Cancer. 2017 Jan 3;17:3. doi: 10.1186/s12885-016-3006-6 (PMC5209874; doi:10.1186/s12885-016-3006-6)

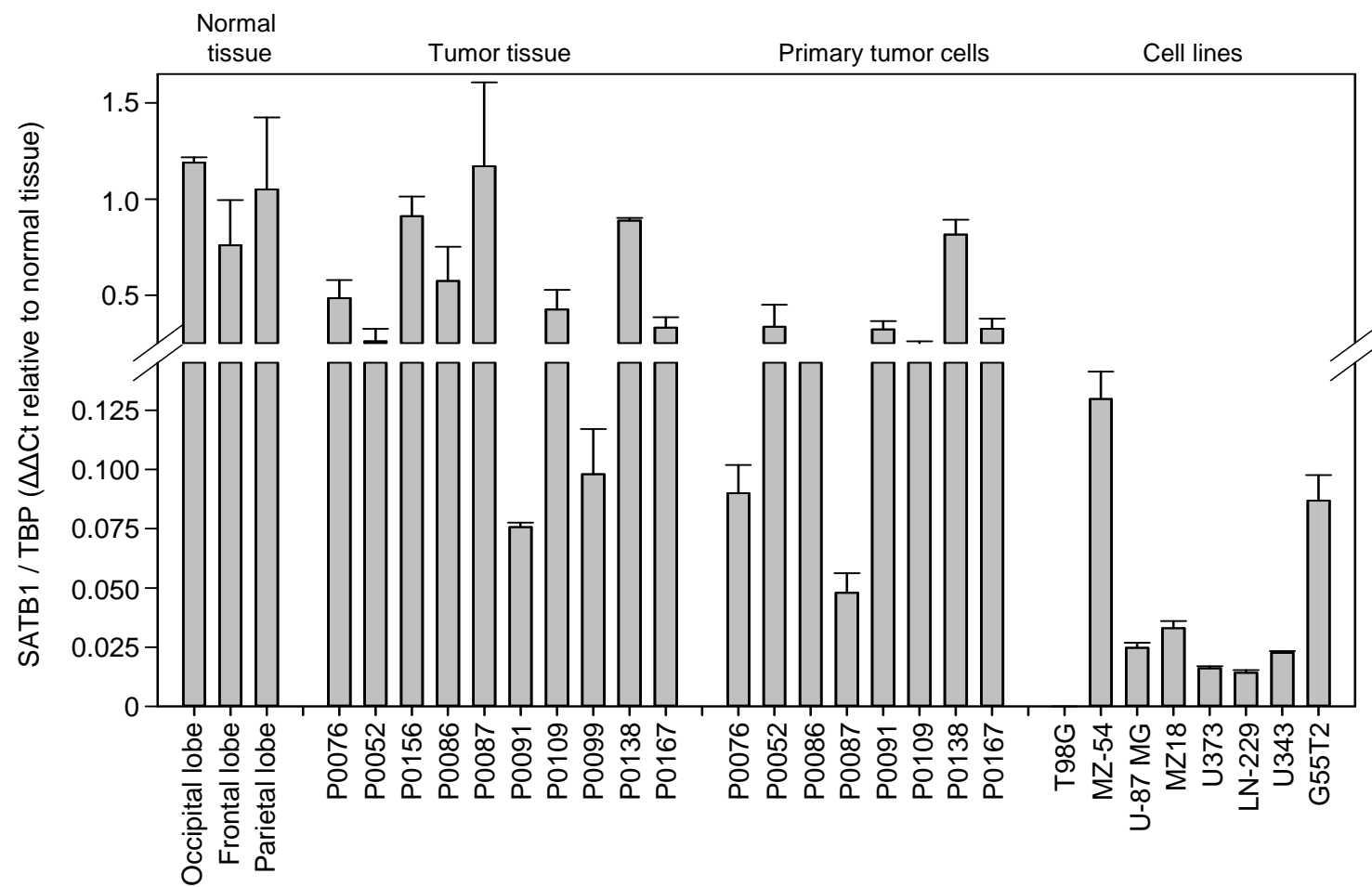

Supplement: Additional file 4: Figure S1. — SATB1 mRNA levels in normal brain tissue versus primary glioblastoma tissue and primary cell lines derived thereof, and established cell lines. Expression levels were determined by qRT-PCR. Denominations of the primary material on the x-axis refer to patients’ IDs. (PDF 12 kb) [file 12885_2016_3006_MOESM4_ESM.pdf]

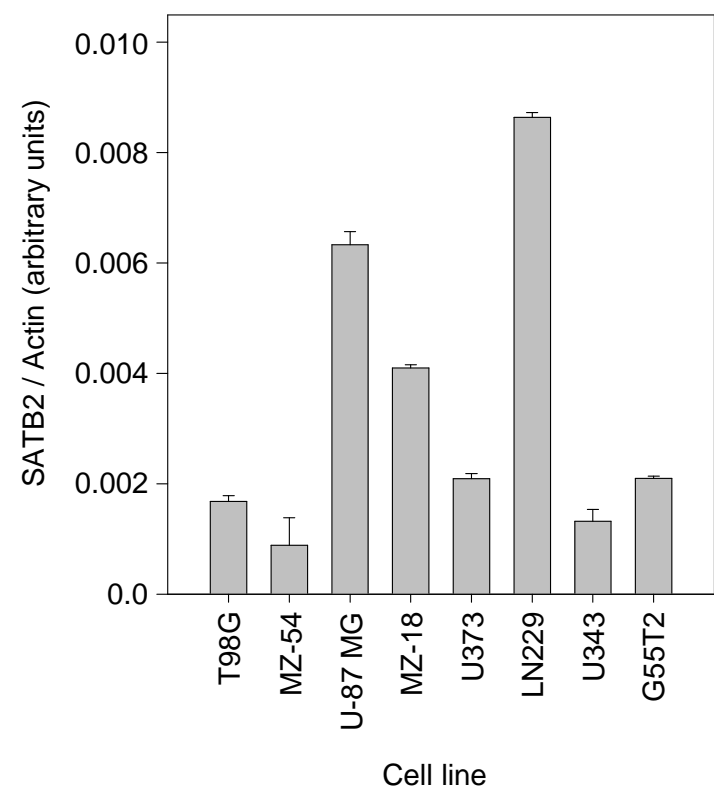

Supplement: Additional file 5: Figure S2. — Expression of SATB2 in various glioblastoma cell lines, as determined on the mRNA level by qRT-PCR. (PDF 4 kb) [file 12885_2016_3006_MOESM5_ESM.pdf]

**A**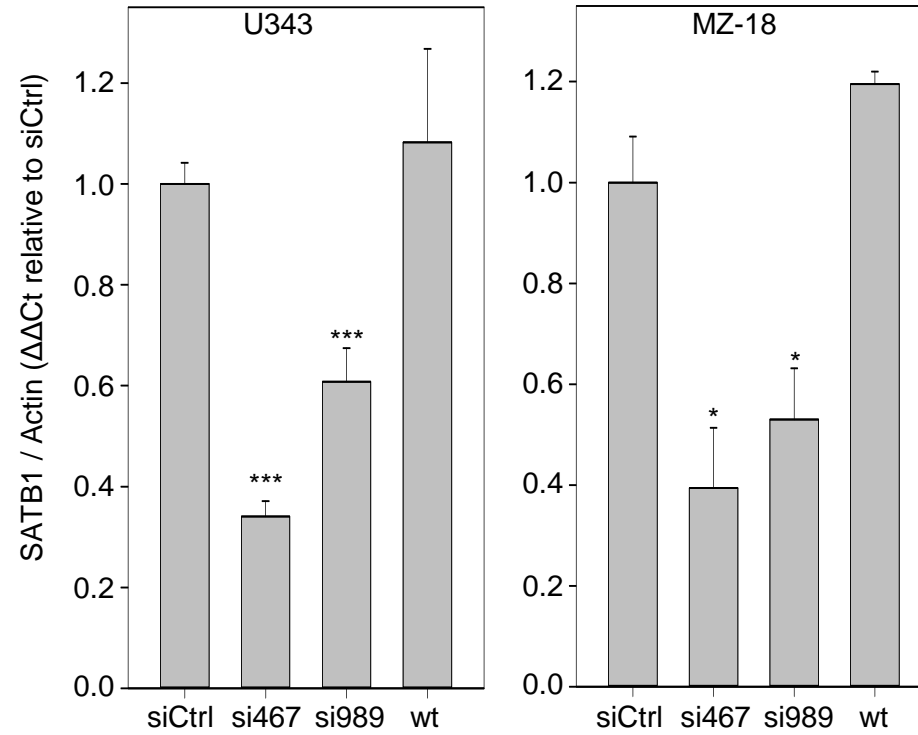**B**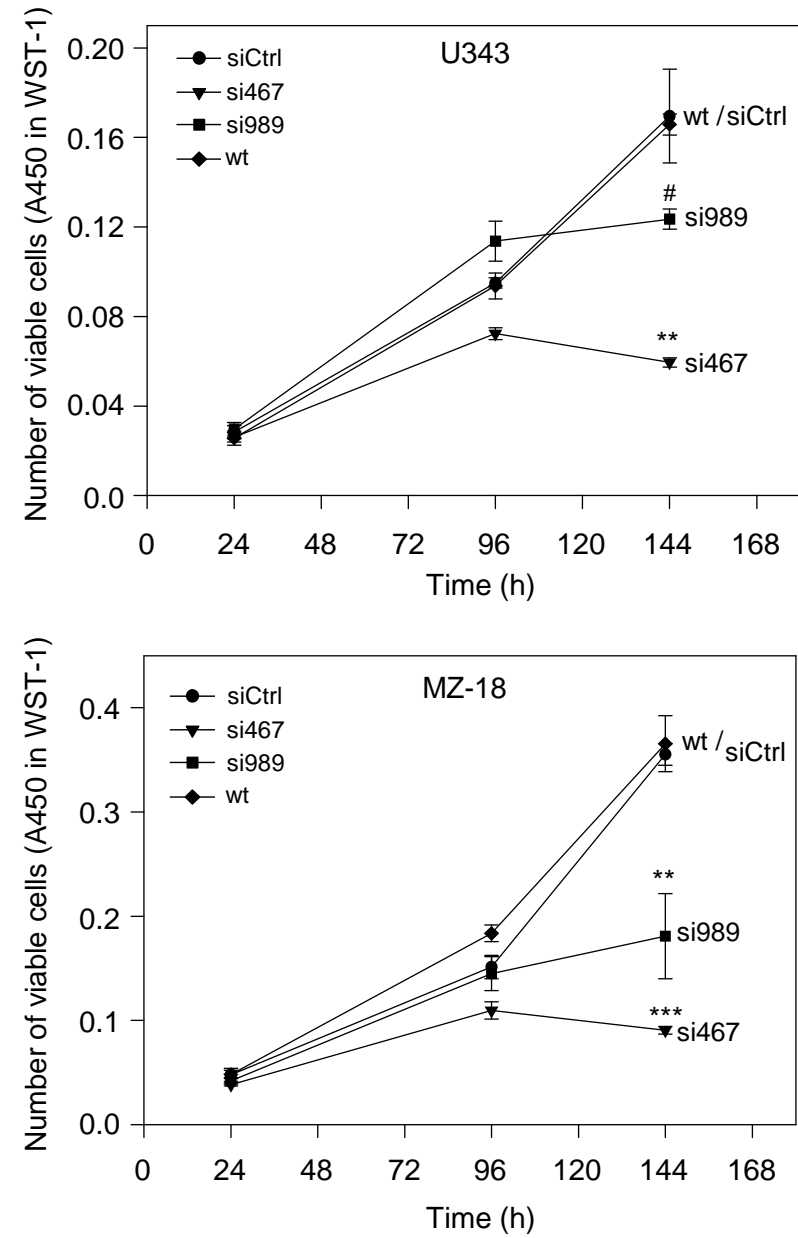

Supplement: Additional file 6: Figure S3. — siRNA-mediated SATB1 knockdown and tumor cell inhibition. (A) SATB1 knockdown upon transfection of SATB1-specific siRNAs si467 or si989 in U343 and MZ-18 glioblastoma cells, as determined on mRNA level (n = 2–3 experiments performed in duplicates and analyzed 72 after transfection). Actin was used as loading control. (B) Marked inhibition of anchorage-dependent proliferation, particularly when using the more potent si467. (PDF 14 kb) [file 12885_2016_3006_MOESM6_ESM.pdf]
